# The leucine biosynthetic pathway is crucial for adaptation to iron starvation and virulence in *Aspergillus fumigatus*

**DOI:** 10.1080/21505594.2019.1682760

**Published:** 2019-11-06

**Authors:** Thomas Orasch, Anna-Maria Dietl, Yana Shadkchan, Ulrike Binder, Ingo Bauer, Cornelia Lass-Flörl, Nir Osherov, Hubertus Haas

**Affiliations:** a Institute of Molecular Biology, Medical University of Innsbruck, Innsbruck, Austria; b Aspergillus and Antifungal Research Laboratory, Department of Clinical Microbiology and Immunology, Sackler School of Medicine, Tel Aviv University, Tel-Aviv, Israel; c Institute of Hygiene & Medical Microbiology, Medical University of Innsbruck, Innsbruck, Austria

**Keywords:** *Aspergillus fumigatus*, leucine, virulence, iron, amino acid biosynthesis

## Abstract

In contrast to mammalia, fungi are able to synthesize the branched-chain amino acid leucine *de novo*. Recently, the transcription factor LeuB has been shown to cross-regulate leucine biosynthesis, nitrogen metabolism and iron homeostasis in *Aspergillus fumigatus*, the most common human mold pathogen. Moreover, the leucine biosynthetic pathway intermediate α-isopropylmalate (α-IPM) has previously been shown to posttranslationally activate LeuB homologs in *S. cerevisiae* and *A. nidulans*. Here, we demonstrate that in *A. fumigatus* inactivation of both leucine biosynthetic enzymes α-IPM synthase (LeuC), which disrupts α-IPM synthesis, and α-IPM isomerase (LeuA), which causes cellular α-IPM accumulation, results in leucine auxotrophy. However, compared to lack of LeuA, lack of LeuC resulted in increased leucine dependence, a growth defect during iron starvation and decreased expression of LeuB-regulated genes including genes involved in iron acquisition. Lack of either LeuA or LeuC decreased virulence in an insect infection model, and inactivation of LeuC rendered *A. fumigatus* avirulent in a pulmonary aspergillosis mouse model. Taken together, we demonstrate that the lack of two leucine biosynthetic enzymes, LeuA and LeuC, results in significant phenotypic consequences indicating that the regulator LeuB is activated by α-IPM in *A. fumigatus* and that the leucine biosynthetic pathway is an attractive target for the development of antifungal drugs.

## Introduction

*Aspergillus fumigatus* is a ubiquitous saprophytic mold and, at the same time, the most common mold pathogen in humans causing severe invasive diseases, termed aspergillosis, particularly in patients with a suppressed immune system [1]. Due to the increasing number of people with impaired immunological defense because of medication or underlying diseases, the number of patients suffering from opportunistic fungal infections has risen dramatically over the last 20 years [2,3]. Treatment of invasive aspergillosis (IA) displays various limitations due to increasing resistance, toxicity and the limited spectrum of currently used antifungal substances [4]. Together with the limitations in diagnosing IA, this results in high mortality rates of between 30% and 90% depending on the patient cohort [5]. To address this problem, new modes of antifungal therapy are needed. In this regard, fungal pathways that are both essential for fungal growth and/or virulence and absent in mammals are of particular interest. Consequently, pathways for biosynthesis of amino acids that are not produced by humans are attractive candidates for antifungal development.

**Figure 1.**
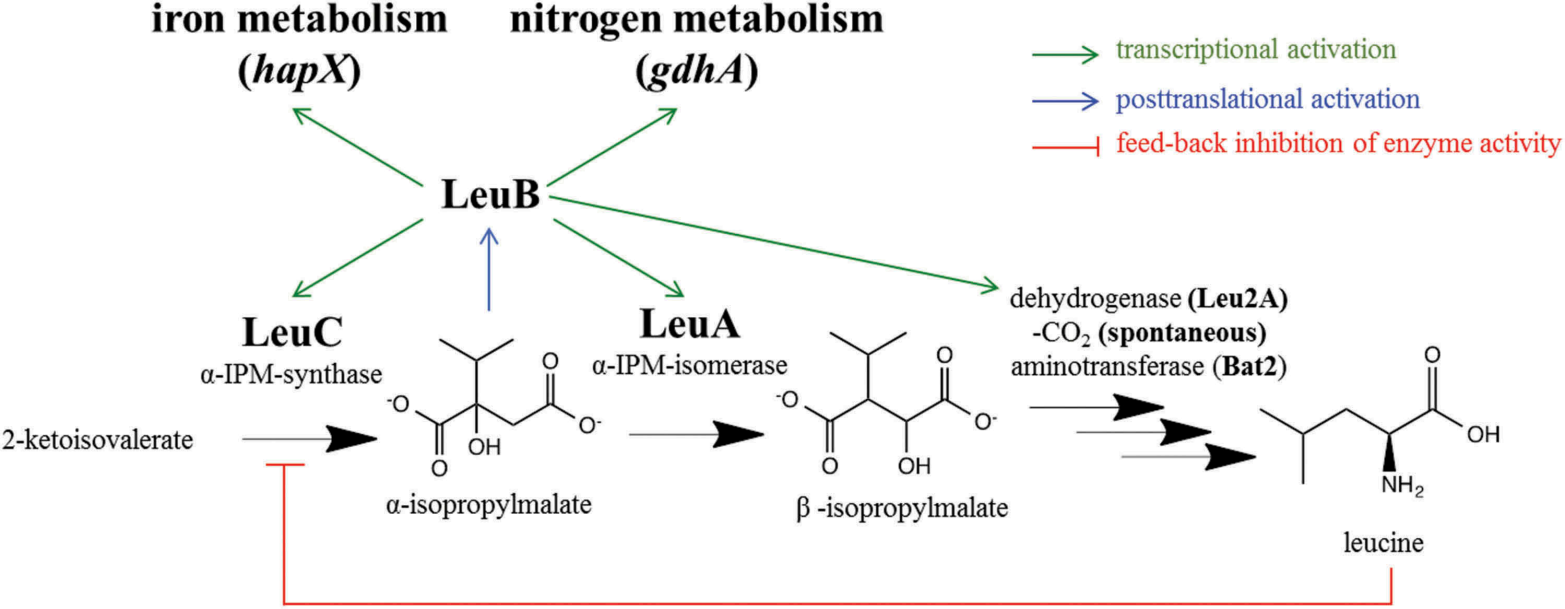
Overview of leucine biosynthesis in *A. fumigatus*. As shown in *S. cerevisiae* [6], 2-ketoisovalerate, a common step of the biosynthesis of valine and leucine, is converted by LeuC (α-IPM synthase) to α-IPM. LeuA (α-IPM isomerase) produces β-IPM, which is subsequently converted by Leu2A (dehydrogenase), spontaneous decarboxylation and Bat2 (BCAA aminotransferase) to leucine. The biosynthesis is feedback-inhibited via blocking of LeuC enzymatic activity by the end-product leucine [6]. The intermediate α-IPM was shown to posttranslationally activate LeuB in *S. cerevisiae* and *A. nidulans* [6,7]. The results of this study indicate that α-IPM also activates LeuB in *A. fumigatus* including the regulation of BCAA encoding genes, *gdhA*, and genes involved in adaptation to iron starvation.

The branched-chain amino acids (BCAA) leucine, isoleucine, and valine are synthesized by bacteria, archaebacteria and eukaryotes such as plants and fungi. In contrast, mammalia do not have the capacity to synthesize BCAAs and satisfy their needs by uptake from the diet. BCAA biosynthesis has three common steps until the production of 2-ketoisovalerate and subsequently splits in the synthesis of valine, leucine, and isoleucine. In *Saccharomyces cerevisiae* [6], leucine-specific biosynthesis starts with the conversion of 2-ketoisovalerate to α-isopropylmalate (α-IPM) by the α-IPM synthase LeuC (*S. cerevisiae* contains two paralogs Leu4p and Leu9p). α-IPM is then further processed by the α-IPM isomerase (Leu1p), the β-isopropylmalate (β-IPM) dehydrogenase (Leu2p), and the branched-chain amino acid aminotransferase (Bat2p). Leucine biosynthesis is feedback regulated *via* inhibition of Leu4p enzyme activity by leucine. Moreover, in *S. cerevisiae* the pathway intermediate α-IPM has been shown to posttranslationally activate the transcription factor Leu3p, which activates several steps in leucine biosynthesis as well as the NADP-dependent glutamate dehydrogenase Gdh1p involved in nitrogen metabolism [6]. Likewise, the Leu3p orthologue LeuB transcriptionally activates genes involved in leucine biosynthesis as well as the glutamate dehydrogenase-encoding gene *gdhA* upon posttranslational activation by α-IPM, in *Aspergillus nidulans* [7]. Recent studies revealed that LeuB additionally regulates iron acquisition in *A. fumigatus* via transcriptional activation of the genes encoding the iron regulator HapX as well as siderophore biosynthetic enzymes [8]. HapX is the major regulator for adaptation to iron starvation in *A. fumigatus* and stimulates the expression of genes involved in iron uptake, including the siderophore system, and represses genes involved in iron consuming pathways, like the tricarboxylic acid (TCA) cycle, respiration or heme biosynthesis [9]. The link to iron homeostasis might be explained by the fact that two BCAA biosynthetic enzymes, Ilv3 and LeuA, require iron-sulfur clusters as prosthetic groups, i.e. BCAA biosynthesis is iron-dependent and requires increased iron accumulation. Moreover, HapX has been shown to transcriptionally repress LeuA and Ilv3A during iron starvation [10]. An overview of leucine-specific biosynthesis in *S. cerevisiae* is shown in Figure S1 and the final proposed steps in *A. fumigatus*, supported by BLAST searches, are shown in Figure 1.

The importance of the early steps in BCAA biosynthesis for virulence has been studied in different fungi. Inactivation of the first common step in BCAA biosynthesis, the acetolactate synthase (termed Ilv2), resulted in decreased virulence in *Cryptococcus neoformans* and *Candida albicans* as well as reduced *in vivo* persistence of *S. cerevisiae* [11–13]. Moreover, the third common step, the dihydroxyacid dehydratase was shown to play a crucial role in virulence of *A. fumigatus*, whereby this mold possesses four potential paralogs, termed Ilv3A, Ilv3B, Ilv3C and Ilv3D [14].

In this study, we investigated the role of the leucine biosynthesis in iron homeostasis and virulence of *A. fumigatus* via deletion of the genes encoding the biosynthetic enzymes LeuC and LeuA, and analyzed the potential regulatory role of α-IPM in *A. fumigatus*.

## Materials and methods

### Media & growth conditions

*A. fumigatus* strains were grown at 37°C on complete medium (2 g/L peptone and 1g/L yeast extract), on blood agar (containing 25% blood) or on/in *Aspergillus* minimal medium [15], containing 20 mM glutamine (nitrogen source), 1% (w/v) glucose (carbon source) and 30 µM FeSO_4_. For iron starvation conditions, the addition of FeSO_4_ was omitted, and for harsher iron starvation conditions on solid media, BPS (bathophenanthrolinedisulfonic acid disodium salt; Sigma-Aldrich, Vienna, Austria), a ferrous iron chelator, was added. All other supplements are described in the respective experiments. In liquid cultures, 10^6^ spores/mL medium were inoculated; and on solid media 10^3^ spores were point-inoculated.

### A. fumigatus strains, deletion of leuA and leuC and reconstitution of ∆leuA and ∆leuC

To generate the mutant strains lacking the leucine biosynthetic enzymes LeuA (AFUB_027020) or LeuC (AFUB_014560), the bipartite marker technique was used [16]. The fragments of the incomplete but overlapping hygromycin (*hph*) resistance cassette were fused to 1 kb of the 5ʹ- and 3ʹ- flanking region of *leuA* or *leuC*, respectively, and transformed into the *A. fumigatus* wildtype (wt) strain A1160P+, resulting in the mutant strains *∆leuA* and *∆leuC*. The flanking regions and the *hph* resistance cassette were PCR-amplified from genomic A1160P+ DNA or the plasmid pAN7.1 using the primer pairs leuA5ʹFW+/leuA5ʹRV+, leuA3ʹFW+/leuA3ʹRV+ and leuAhphFW/leuAhphRV for *leuA* and TO3/TO4, leuC3ʹFW+/leuC3ʹRV+ and TO15/leuChphRV for *leuC*, respectively. Together with the pUC19L vector (containing an ampicillin resistance cassette), the fragments were assembled with the GeneArt Seamless PLUS Cloning and Assembly Kit (Thermo Fisher Scientific, Vienna, Austria) resulting in the plasmids p∆leuA and p∆leuC. Deletion constructs for transformation were amplified with the primers leuA5ʹFW/ohph14 (2.3 kb) and leuA3ʹRV/ohph15 (2.5 kb) or TO14/ohph14 (2.3 kb) and leuC3ʹRV/ohph15 (2.5 kb). Protoplasts were transformed with the two split marker fragments for each gene and the mutant strains were selected on minimal media containing 0.1 mg/mL hygromycin B (Calbiochem).

To reconstitute *leuA* and *leuC* in the deletion mutants, fragments containing a complete *leuA* (4.4 kb) or *leuC* (4.3 kb) copy were amplified from genomic A1160P+ DNA with the primer pairs TO52/TO53 for *leuA* and TO54/TO55 for *leuC*, respectively, and subsequent to gel purification, the fragments were digested with *Hind*III and *Nde*I (*leuA*) or *Not*I and *Spe*I (*leuC*). The plasmid pSK275 carrying the pyrithiamine resistance cassette (*ptrA*) was digested with the respective enzymes and ligated with the *leuA* or *leuC* fragment, resulting in the plasmids pleuA^rec^ and pleuC^rec^. These plasmids were linearized by digestion with *Avr*II (pleuA^rec^) or *Hpa*I (pleuC^rec^) to promote homologous recombination in the 5ʹ-flanking regions of *leuA* or *leuC* and integrated into the deletion locus of the corresponding leucine biosynthetic enzyme by protoplast transformation. The reconstituted strains were selected with 0.1 µg/mL pyrithiamine hydrobromide and for leucine prototrophy. The correct integration of all constructs was verified by Southern blot analysis (data not shown). An overview of the deletion strategy is shown in Figure S2. All strains used in this study are listed in Table S1. Primers used for genetic manipulation are shown in Table S2.

### RNA isolation and northern blot analysis

Fungal strains were cultured in the liquid minimal medium under iron starvation conditions at 37°C and 200 rpm with or without the addition of 5 mM leucine as described in the Figure legends. RNA isolation was performed using TRI Reagent® (Sigma-Aldrich, Vienna, Austria) following the manufacturer’s manual. Ten micrograms of RNA (if not indicated otherwise) were loaded on 2.2 M formaldehyde agarose gels and blotted after electrophoresis onto Amersham Hybond N membranes (GE Healthcare, Vienna, Austria). RNA was detected with DIG-labeled probes, amplified by PCR. Primers used for these probes are shown in Table S2.

### Southern blot analysis

Genomic DNA was isolated according to Sambrook et al. [17]. To confirm the correct integration of the genetic manipulations, the DNA was digested with*Bam*HI (∆*leuA, leuA*^rec^, wt) or *Pvu*II (∆*leuC*, *leuC*^rec^, wt), separated on an agarose gels and blotted onto Amersham Hybond N membranes (GE Healthcare, Vienna, Austria). The different fragment sizes were detected with DIG-labeled probes of the 5ʹ- and 3ʹ-flanks of the corresponding gene. Primers used for these probes are shown in Table S2.

### Quantification of siderophore production

Extracellular siderophores were extracted from culture supernatants and detection was performed as described previously [10].

### *Virulence assay in* Galleria mellonella

Virulence studies in the *G. mellonella* infection model were performed according to Fallon et al. [18]. Larvae of the greater wax-moth *G. mellonella* (SAGIP, Italy) were kept at 18°C in the dark before use to prevent pupation. Randomly selected larvae, weighing between 0.3 and 0.4 g, were injected through one of the hind pro-legs with 10^7^ spores of *A. fumigatus* wt, ∆*leuA, leuA*^rec^, ∆*leuC,* or *leuC*^rec^ in 20 µL insect physiological saline (IPS) into the hemocoel. As controls, untreated larvae and larvae injected with IPS were used. All larvae were incubated at 30°C in the dark, to circumvent temperature-related immune response of the larvae as shown previously [19], and viability was monitored for 6 days post infection. Experiments were repeated three times with groups of 20 larvae per sample each time. Survival data representing the average survival rates of all experiments (total of 60 larvae per sample) were evaluated using Kaplan-Meier curves and analyzed with the log-rank (Mantel-Cox) test utilizing GraphPad Prism 7.00 software. Differences were considered significant at *p* ≤ 0.05.

### Virulence testing in a pulmonary mouse model

A non-neutropenic mouse model was used for virulence testing. Therefore, six-week-old female ICR mice (10 per group) were immunocompromised by subcutaneous injection of 300 mg/kg body weight cortisone acetate on day −3, 0, 3, 7 and 11 of infection. For pulmonary infection, a suspension of 5 × 10^5^ spores of wt, ∆*leuC* or *leuC^rec^* in 20 µL PBS plus 0.2% Tween 20 was applied intranasally (10 µL in each mouse nostril). Survival was observed for 21 days post infection and analysis of the survival data was performed by Kaplan-Meier survival analysis. Lung fungal burden was assessed by infecting immunocompromised mice (n = 5) as described above, sacrificing them 48 h postinfection, homogenizing the lungs and plating dilutions on YAG plates for CFU (colony forming unit) enumeration. Three mice in the wt group and two in the *leuC^rec^* group were found moribund before completion of the 48 h infection and were therefore not included in the analysis. Histology was performed with gomori methenamine silver stain (GMS, stains fungal elements black) or haematoxylin and eosin stain (H&E, stains host-cell nuclei purple, cytosol pink).

The Ministry of Health (MOH) Animal Welfare Committee, Israel, ethically approved these experiments.

## Results

### Lack of leuC results in higher leucine requirement for growth compared to lack of leuA

Homologues of leucine biosynthetic and regulatory enzymes in *A. fumigatus* were identified by BLAST analysis (Table S3). In contrast to *S. cerevisiae*, which contains two α-IPM synthase homologs (termed Leu4 and Leu9), *A. fumigatus* possesses only a single enzyme, termed LeuC (AFUB_014560); the α-IPM isomerase (in *S. cerevisiae* LEU1p) was termed LeuA (AFUB_027020), the β-isopropylmalate dehydrogenase (in *S. cerevisiae* Leu2p) was termed Leu2A (AFUB_015310) and the homolog of the transcription factor Leu3p is termed LeuB (AFUB_020530).

To investigate the role of leucine biosynthesis in *A. fumigatus*, we generated two mutant strains lacking either the α-IPM isomerase LeuA (strain ∆*leuA*) or the α-IPM synthase LeuC (strain ∆*leuC*) by replacing the coding region of the respective gene with the hygromycin resistance cassette. A single copy of the respective gene was reintegrated in the same locus yielding the strains *leuA^rec^* and *leuC^rec^* to ensure that the phenotypic consequences observed are indeed due to the gene deletions. Correct genetic integration was confirmed by Southern blot analysis.

**Figure 2.**
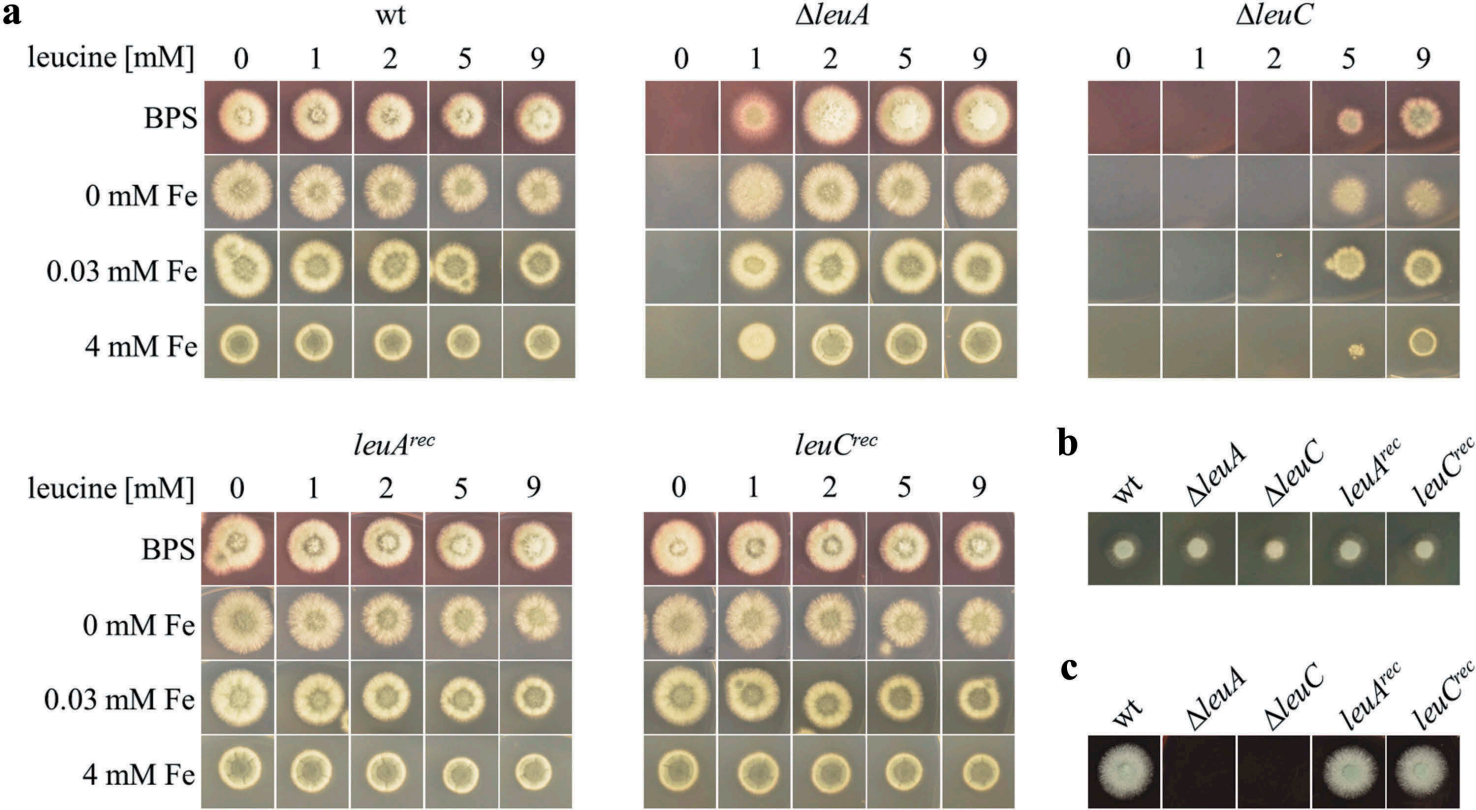
Strains lacking LeuA or LeuC display leucine auxotrophy and ∆*leuC* requires higher leucine supplementation for growth. Growth of ∆*leuA* and ∆*leuC* compared to wt and the reconstituted strains was analyzed on minimal medium (a) complete medium (b) or 25% blood agar (c). Generally, 10^3^ spores were point-inoculated on the respective solid growth medium and incubated at 37°C. Minimal medium contained different concentrations of ferrous iron (0 mM, 0.03 mM, or 4 mM), the ferrous iron-specific chelator BPS, and/or leucine (0 mM, 1 mM, 2 mM, 5 mM, or 9 mM) as indicated. 0.03 mM iron reflects the standard minimal medium. Growth was evaluated after incubation for 48 h on minimal medium, 24 h on complete medium and 36 h on blood agar. Representatives of at least three biological replicates are shown.

Growth analysis of all strains on plates (Figure 2(a)) revealed leucine auxotrophy of both the ∆*leuA* and ∆*leuC* strain, which was cured by the reintegration of the respective gene resulting in *leuA^rec^* and *leuC^rec^* strains. Remarkably, the ∆*leuC* mutant required a significantly higher leucine supplementation for growth compared to the ∆*leuA* mutant, i.e. on minimal medium plate cultures, ∆*leuA* was able to grow with 1 mM leucine supplementation while the ∆*leuC* mutant required ≥5 mM (Figure 2(a)). Nine millimolar leucine supplementation did improve the growth of the ∆*leuC* mutant but did not fully reconstitute wt-like growth. Supplementation with the leucine biosynthesis intermediates α-IPM or β-IPM did not rescue growth of the ∆*leuA* or ∆*leuC* mutants (Figure S3), which indicates that α-IPM or β-IPM are not taken up efficiently by *A. fumigatus*. As leucine biosynthesis is dependent on iron and as the leucine biosynthesis regulator LeuB was recently found to be involved in the regulation of iron acquisition [8], we examined the effect of iron availability on the growth of the two leucine auxotrophic strains. Supplementation with different iron concentrations or the presence of the ferrous iron-specific chelator BPS, which generates iron starvation [20], did not significantly affect the leucine requirement of both mutants on plates (Figure 2(a)). On complete medium containing yeast extract and peptone, which both contain leucine, both mutant strains showed wt-like growth (Figure 2(b)). In contrast, on blood agar plates, neither ∆*leuA* nor ∆*leuC* was able to grow (Figure 2(c)).

Taken together, the deletion of either *leuA* or *leuC* causes leucine auxotrophy, demonstrating that there are no other genes able to compensate for their loss. The difference in the degree of leucine requirement for growth between the two leucine auxotrophic strains indicates a regulatory role of α-IPM, which is missing in ∆*leuC* but accumulating in ∆*leuA* (Figure 1).

### Lack of leuC impairs growth particularly during iron starvation

**Figure 3.**
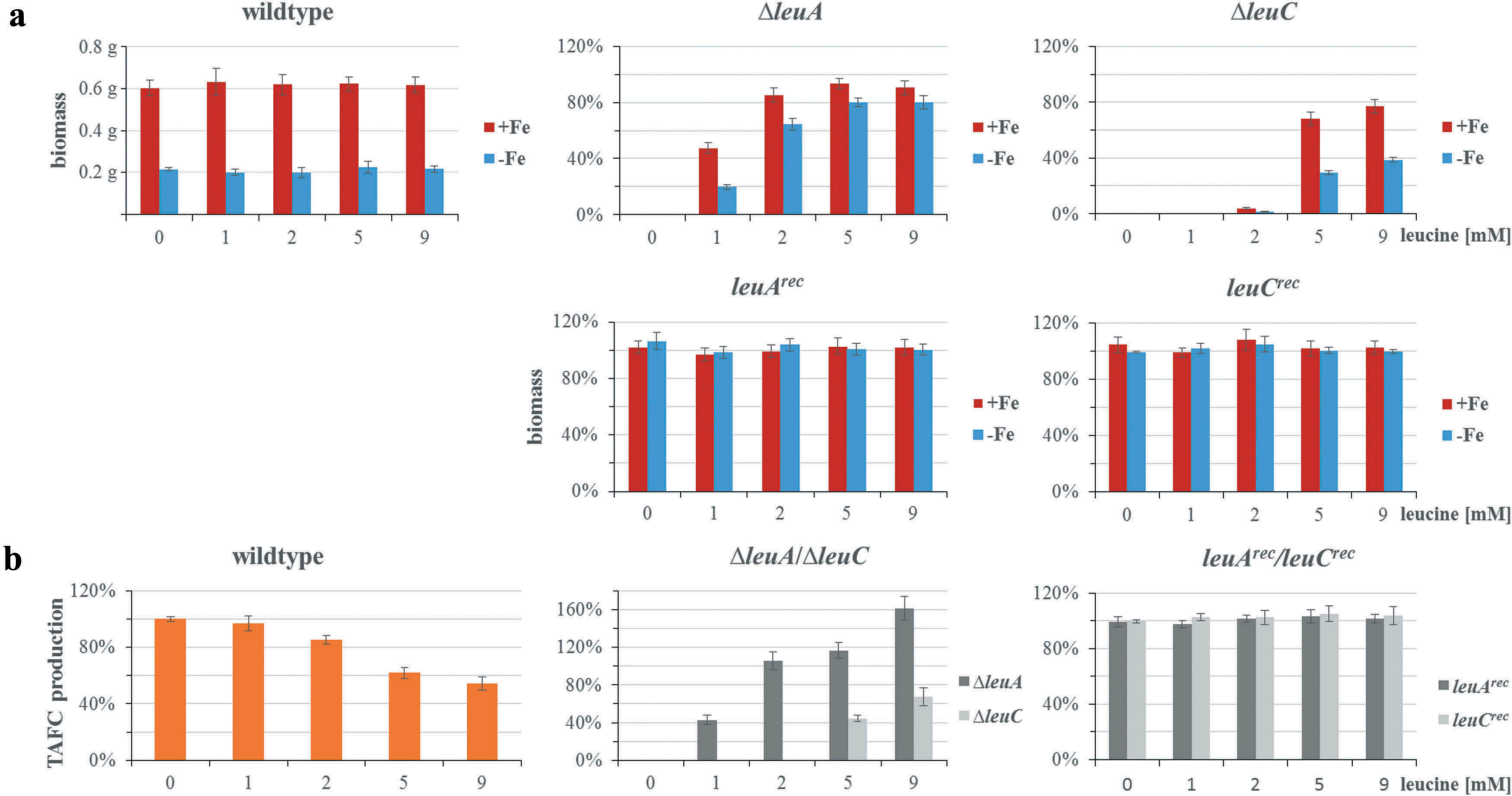
Lack of LeuC results in decreased growth and TAFC production in iron-limited liquid cultures. 10^6^ spores/mL of the respective strains were cultured in 100 mL liquid minimal medium containing 0, 1, 2, 5 or 9 mM of leucine with or without addition of 0.03 mM of FeSO_4_ at 37°C for 30 h. (a) For the wt, the absolute biomass is displayed, while for the deletion and reconstituted strains, the biomass is normalized to the wt biomass under the same conditions. (b) TAFC content of the culture supernatants was normalized to the respective biomass. TAFC production of the wt is shown normalized to that without leucine supplementation, while TAFC production of the deletion and reconstituted mutant strains is shown normalized to the wt production at the same leucine concentration. The data show the mean ± standard deviation of three biological replicates.

Similar to plate growth assays, the ∆*leuC* mutant required significantly higher leucine supplementation for growth compared to the ∆*leuA* mutant in liquid growth conditions (Figure 3(a)), i.e. under standard iron sufficient conditions (30 µM FeSO_4_) with 1 mM leucine supplementation, ∆*leuA* reached about 50% of the wt biomass, while ∆*leuC* did not grow. In contrast, growth of wt, *leuA^rec^*, and *leuC^rec^* in liquid cultures was not affected by leucine supplementation, neither under iron starvation nor under iron sufficient conditions (Figure 3(a)).

Iron limitation reduced biomass formation of wt and reconstituted mutant strains to about 33% compared to iron sufficiency. With 9 mM leucine supplementation, the ∆*leuA* mutant produced 90% of the wt biomass during iron sufficiency and 80% of the wt biomass during iron starvation. In comparison, with 9 mM leucine supplementation, the ∆*leuC* mutant produced 78% of the wt biomass during iron sufficiency, but only 39% of the wt biomass during iron starvation. In other words, with 9 mM leucine supplementation, ∆*leuC* produced 87% of the ∆*leuA* biomass during iron sufficiency but only 49% of the ∆*leuA* biomass during iron starvation, which indicates a particular growth defect during iron starvation.

### Siderophore production is repressed by leucine supplementation and lack of leuC

Production of the extracellular siderophore triacetylfusarinine C (TAFC) was decreased in wt in a leucine concentration-dependent manner with 9 mM leucine reducing TAFC production to 54% of that without leucine supplementation (Figure 3(b)).

The ∆*leuA* strain displayed wt-like TAFC production at 2 mM and 5 mM leucine and exceeded the wt by 1.6-fold at the same leucine supplementation of 9 mM leucine (Figure 3(b)). In contrast, the ∆*leuC* mutant strain displayed significantly reduced TAFC production, i.e. compared to wt, TAFC production was decreased to 45% and 68% with 5 mM and 9 mM leucine supplementation, respectively. Taken together, the ∆*leuC* mutant displayed significantly decreased (>50%) TAFC production compared to the ∆*leuA* strain (Figure 3(b)). The reconstituted mutant strains displayed wt-like TAFC production, confirming that these effects are indeed caused by the gene deletions.

### Lack of leuC but not of leuA impairs activation of leuB target genes involved in leucine biosynthesis, nitrogen metabolism, and iron acquisition

**Figure 4.**
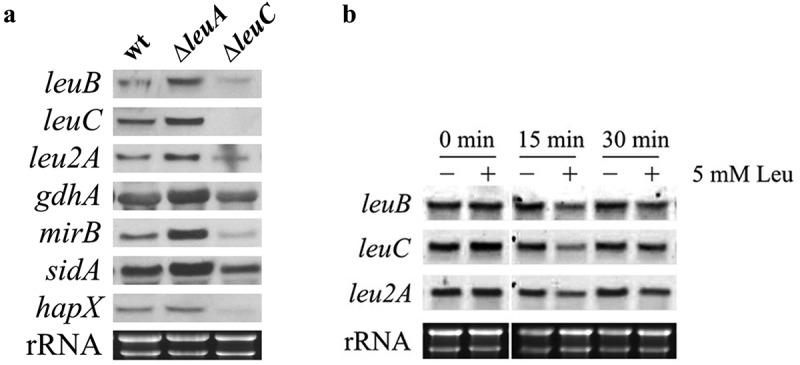
Transcript levels of *leuB* and LeuB target genes are upregulated in ∆*leuA* but downregulated in ∆*leuC* compared to wt (a) and downregulated by leucine supplementation in wt (b). (a) For Northern blot analysis, fungal strains were cultured under iron starvation conditions with 5 mM leucine supplementation. The wt strain was grown for 16 h, ∆*leuA* for 20 h, and ∆*leuC* for 28 h to compensate for the different growth rate and to reach the same biomass. (b) To analyze potential autoregulation of LeuB, the wt strain was cultured for 16 h under iron starvation prior the addition of leucine to a final concentration of 5 mM. For Northern analysis, samples were taken at 0, 15 and 30 min after leucine addition.

To assess the potential impact of α-IPM on LeuB-mediated regulation [8], we analyzed the transcript levels of *leuB*, and recently identified LeuB target genes [8] in the wt, *∆leuA*, and *∆leuC* strains grown under iron starvation conditions with 5 mM leucine supplementation by Northern analysis (Figure 4(a)). Compared to LeuA inactivation, LeuC inactivation resulted in strongly decreased transcript levels of *leuB, leu2A, gdhA, mirB* (encoding a transporter for uptake of ferric TAFC), *sidA* (encoding a siderophore biosynthetic gene), and *hapX* (encoding a regulator for adaptation to iron starvation). Compared to wt, lack of LeuA resulted in increased transcript levels of these genes, except *hapX*, also including *leuC*, which is not expressed in *∆leuC* due to the deletion of the gene. The reintegration of the *leuC* gene in *∆leuC* (strain *leuC^rec^*) cured the defect in gene regulation (Figure S4).

The downregulation of *leuB* in *∆leuC* compared to *∆leuA* indicates transcriptional autoregulation of LeuB (Figure 4(a)). This autoregulation is further supported by the fact that supplementation of liquid culture with leucine, which inhibits the enzymatic activity of LeuC (Figure 1), to a final concentration of 5 mM resulted in downregulation of *leuB, leu2A*, and *leuC* within 15 min (Figure 4(b)).

### Lack of leuA or leuC attenuates virulence in the *Galleria mellonella* infection model

**Figure 5.**
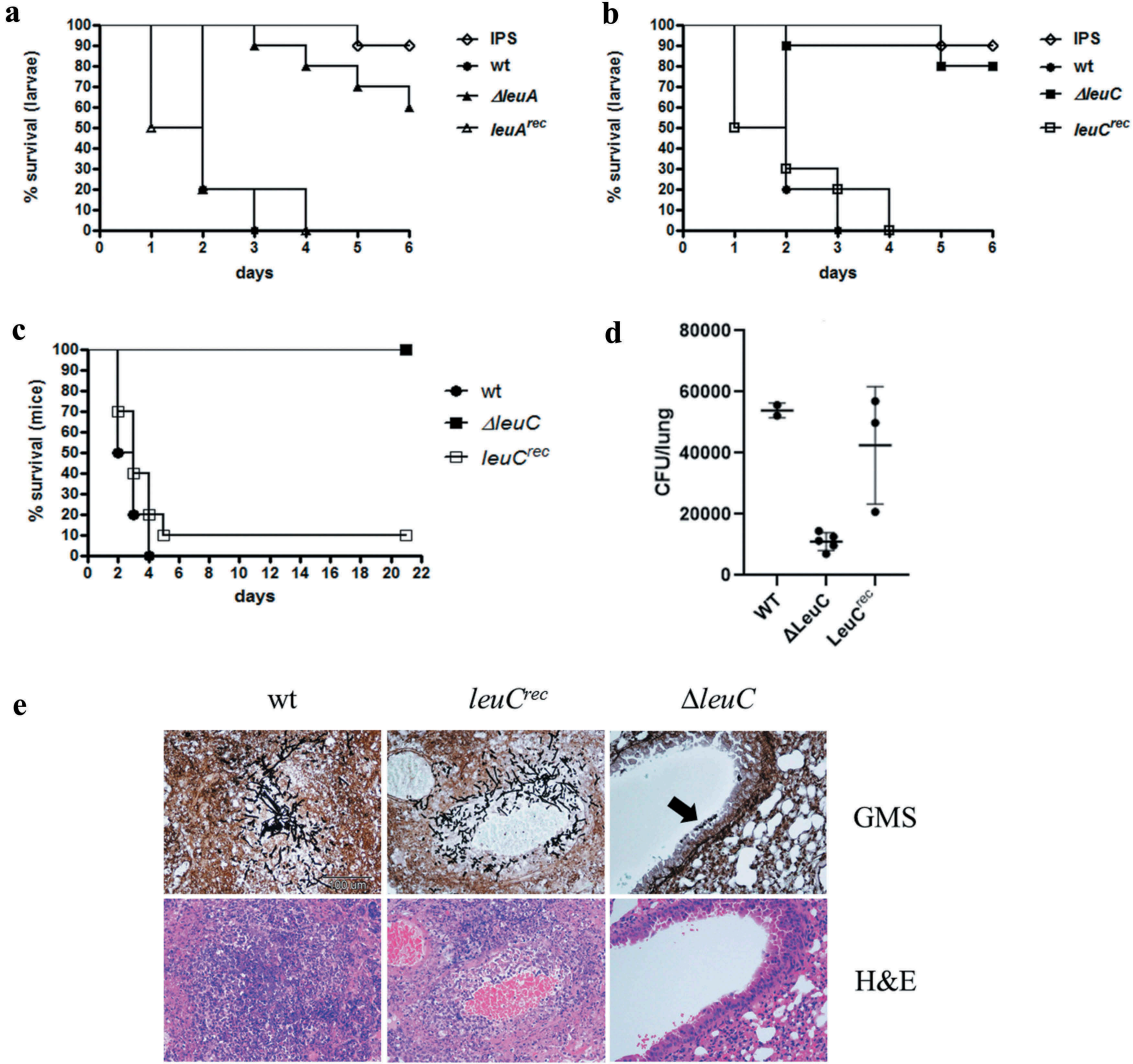
∆*leuA* and ∆*leuC* mutants display attenuated virulence in *Galleria mellonella* and ∆*leuC* is avirulent in a pulmonary aspergillosis mouse model. In *G. mellonella*, ∆*leuA* (a) and ∆*leuC* (b) showed significantly increased survival rates compared to wt (Log-Rank test, p < 0.0001), while survival of the reconstituted strains (*leuA^rec^* and *leuC^rec^*) was statistically indifferent to the wt (p = 0.80 and 0.56, respectively). The curves display the average survival of three independent experiments (in total 60 larvae per sample). (c) In the mouse model (10 mice per strain), the *leuC* deletion strain was avirulent compared to the wt and the reconstituted strain (p < 0.0001). (d) Mouse lung fungal burden is greatly reduced in mice infected with the LeuC-deletion strain compared to the wt (p < 0.0001) and reconstituted strain (p < 0.008). (e) Histological analysis of infected lungs stained with GMS (gomori methenamine silver, stains fungi black, upper panel) shows large foci containing numerous invasive hyphae in mice infected with the wt and reconstituted strain. In contrast, only small infrequent aggregates of swollen conidia were seen in lung sections of mice infected with the ∆*leuC* strain (black arrow, upper panel on the right). Staining of lung cells with H&E shows granulocyte accumulation around the wt and *leuC^rec^ A. fumigatus* strains, but not the ∆*leuC* strain (lower panel).

To investigate the influence of leucine biosynthesis on *A. fumigatus* infections, we compared the virulence potential of wt, *∆leuA* and *∆leuC* in the *G. mellonella* wax-moth infection model. Lack of LeuA or LeuC resulted in significantly higher survival rates (p < 0.0001) of the larvae compared to wt over the examined period of 6 days (Figure 5(a,b)). While all larvae infected with wt spores died on day 3, only 10% of *∆leuA* and *∆leuC* infected larvae were dead. By the end of the experiment (day 6) survival rates of *ΔleuA* and *ΔleuC* still were 60% and 80%, respectively. Untouched larvae showed 100% survival (data not shown) and reintegration of the deleted genes (strains *leuA^rec^* and *leuC^rec^*) cured the virulence defect (Figure 5(a,b)). Taken together, these data indicate a crucial role for leucine biosynthesis in the virulence of *A. fumigatus*.

### Lack of leuC results in attenuated virulence in a pulmonary aspergillosis mouse model

LeuA has a homolog in humans, the aconitase 2, while LeuC shows no significant similarity to human proteins. Moreover, LeuC-deletion resulted in a more severe growth defect compared to the deletion of LeuA. Therefore, only the *∆leuC* mutant strain was used to assess the role of leucine biosynthesis in a murine pulmonary aspergillosis model. Mice infected with wt or *leuC^rec^* displayed high mortality, resulting in survival rates of 0% (wt) and 10% (*leuC^rec^*). In contrast, the *leuC* deletion strain demonstrated total avirulence (p < 0.0001) compared to the wt and the reconstituted strain with all the mice surviving over the examined period of 21 days (Figure 5(c)). The lung fungal burden of mice infected with the *∆leuC* mutant strain was significantly reduced in comparison to those of mice infected with wt (p < 0.0001) and *leuC^rec^*(p < 0.008) strains (Figure 5(d)). Histological examination of mouse lungs showed greatly reduced fungal growth and inflammatory cell influx for mice infected with the *∆leuC* mutant strain compared to those infected with the wt or *leuC^rec^* strains (Figure 5(e)).

## Discussion

The BCAAs leucine, valine, and isoleucine are produced exclusively by bacteria, archaebacteria and lower eukaryotes including plants and fungi [6]. Mammals lack these biosynthetic pathways and consequently have to satisfy the need for these essential amino acids by nutritional uptake. This makes the BCAA biosynthetic pathway an attractive target for new antifungal agents unless the concentration of the amino acids in the host niche is high enough to cure the auxotrophy caused by inhibition of the targeted enzyme. Compounds targeting the first step of BCAA biosynthesis, the acetolactate synthase (termed Ilv2 in *S. cerevisiae*), are already used as herbicides [21]. Moreover, the deletion of the gene encoding this enzyme decreased *in vivo* persistence of *S. cerevisiae* [11] and attenuated virulence of *C. neoformans* and *C. albicans* in murine models [12,13]. However, antifungal activity of currently available Ilv2 inhibitors, such as triazolopyrimidine-sulfonamide compounds, can be bypassed through supplementation with BCAA or serum, which limits their application for treatment of mammalian infections [22]. Deletion of the third BCAA biosynthesis step, the dihydroxyacid dehydratase (termed Ilv3A), was shown to attenuate virulence in *A. fumigatus* [14]. Interestingly, the latter study revealed that the growth defect of dihydroxyacid dehydratase-lacking strains can be restored by valine and isoleucine supplementation, while leucine supplementation had a negative influence on the growth [14]. Focusing on leucine biosynthesis alone, lack of the LeuA homolog Leu1 was shown to attenuate virulence in a pulmonary mouse model of *C. neoformans* [23]. Interestingly, lack of the Leu1 homolog was also found to increase abundance of two mitochondrial iron-sulfur cluster proteins (aconitase and the iron-sulfur biosynthetic enzyme Nfu1) during iron starvation, but not iron sufficiency, but the mechanism behind this was unclear. In contrast, leucine auxotrophy did not affect *in vivo* persistence of *Candida glabrata* or *C. albicans*, respectively [24,25].

To analyze exclusively the role of leucine biosynthesis in virulence, we focused on two enzymes, LeuA and LeuC (in contrast to *A. fumigatus, S. cerevisiae* employs two homologs termed Leu4 and Leu9). BLAST search revealed that mammals have a LeuA homologous protein, termed aconitase (CAG38805.1; E-value = 9e-26 with 25% identity in 484 amino acids) but lack LeuC homologous proteins.

In this study, we found that lack of either LeuA or LeuC causes leucine auxotrophy in *A. fumigatus*. Lack of either LeuA or LeuB blocked growth on blood agar plates indicating that the leucine content of blood is too low to support the growth of these mutant strains. The latter is in agreement with the reported blood leucine concentration of 0.10–0.14 mM in humans [26].

Remarkably, inactivation of LeuC and LeuA, respectively, resulted in significant differences, i.e. compared to lack of LeuA, lack of LeuC resulted in (i) significantly higher leucine requirement for growth, (ii) decreased growth rate during iron starvation, (iii) decreased siderophore production, and (iv) decreased expression of the gene encoding the NADP-dependent glutamate dehydrogenase, genes involved in leucine biosynthesis, as well as of genes involved in siderophore-mediated iron acquisition.

These data revealed that the inactivation of different steps in the leucine biosynthetic pathway has different phenotypic consequences. The difference between the two mutant strains is that inactivation of LeuC results in the lack of α-IPM, while this pathway intermediate accumulates in LeuA lacking cells. Apart from leucine biosynthesis, other α-IPM-dependent enzymatic pathways are not known. In *S. cerevisiae* and *A. nidulans*, LeuB homologs have been shown to be posttranslationally activated by the leucine biosynthesis intermediate α-IPM, leading to transcriptional activation of LeuB target genes involved in nitrogen metabolism and leucine biosynthesis [6,7]. Recently, LeuB was found to transcriptionally regulate not only leucine biosynthesis and glutamate dehydrogenase but also iron acquisition in *A. fumigatus* and is consequently important for adaptation to iron starvation [8]. The comparison of mutants lacking either LeuA or LeuC offers the possibility to study the regulatory role of α-IPM, as this leucine pathway intermediate is missing in ∆*leuC* but accumulating in ∆*leuA* strains. Inactivation of LeuC had similar consequences as lack of LeuB: a particular growth defect during iron starvation, decreased siderophore production and the decreased expression of the gene encoding glutamate dehydrogenase, genes involved in leucine biosynthesis and genes involved in siderophore-mediated iron acquisition. Taken together, the current data indicate that α-IPM is important for the activation of all currently known functions of LeuB in *A. fumigatus*. The function of the leucine biosynthetic pathway in adaptation to iron starvation is further underlined by the finding that leucine supplementation, which blocks LeuB activity, decreased siderophore production in *A. fumigatus* wt. Consequently, this study reveals for the first time the role of α-IPM in fungal iron homeostasis. The decreased expression of genes involved in siderophore metabolism is in agreement with the reduced siderophore production and explains the reduced growth of the *∆leuC* compared to the *∆leuA* mutant strain during iron starvation. The increased leucine requirement for growth of the *∆leuC* compared to the *∆leuA* mutant strain might indicate that α-IPM-mediated activation of LeuB is required for transcriptional activation of leucine uptake. The leucine permeases of *A. fumigatus* have not been identified yet. BLASTp (https://blast.ncbi.nlm.nih.gov/Blast.cgi) searches revealed that the *A. fumigatus* amino acid permease-encoding gene *gap1* (AFUB_089830) is the *A. fumigatus* amino acid permease with the highest degree of similarity to both *S. cerevisiae* Gap1 (57% identity) and *S. cerevisiae* Bap2 (41% identity), which mediate uptake of leucine in this yeast [27]. The similar *gap1* transcript levels in *wt, leuA, and leuC* mutants suggest that the growth defect of the *leu*C mutant compared to the *leuA* mutant might be due to differences in intracellular leucine handling rather than to leucine uptake.

Northern analysis revealed downregulation of *leuB* in *∆leuC* compared to *∆leuA*, which indicates the transcriptional autoregulation of LeuB. This is supported by the fact that LeuB was previously found to bind to the promoter of its encoding gene *in vivo* [8] and that we found here that leucine supplementation, which inhibits the enzymatic activity of LeuC and thereby activity of LeuB, results in downregulation of *leuB, leu2A*, and *leuC* within 15 min.

In the *Galleria mellonella* infection model, lack of either LeuA or LeuC attenuated virulence of *A. fumigatus*. Consequently, for insect virulence, the defect in leucine biosynthesis appears to be a major reason for the defective virulence. The fact that lack of LeuC caused higher attenuation compared to lack of LeuA is in agreement with the differences in the growth pattern of the respective mutant strains.

In a murine pulmonary aspergillosis model, lack of LeuC resulted in avirulence. This might be the consequence of lacking leucine biosynthesis, the malfunction in adaptation to iron starvation, which has been shown to be important for virulence [10,20], or a combination. In this system, we tested only the virulence of the LeuC-deletion mutant for the following reasons: LeuC-deletion caused a more severe growth defect, particular during iron starvation, which is a known feature of the host niche [28]. Moreover, LeuC lacks mammalian homologs, while LeuA displays significant similarity to aconitase 2. Taken together, LeuC appears to be a more promising target for developing novel antifungal therapies.

In summary, our study shows that (i) inactivation of different components of the same pathway can have significantly different phenotypic consequences, (ii) LeuC may be an attractive target for the development of novel antifungals, and (iii) α-IPM has a regulatory role in *A. fumigatus* similar to *S. cerevisiae* and *A. nidulans*, whereby a role in fungal iron homeostasis is shown for the first time here.

## Abbreviations

α-IPM: α-isopropylmalate
β-IPM: β-isopropylmalate
BCAA: branched chain amino acid
BPS: bathophenanthrolinedisulfonic acid disodium salt
IA: invasive aspergillosis
IPS: insect physiological saline
TAFC: triacetylfusarinine C
wt: wildtype
