# Supplementary material for: The leucine biosynthetic pathway is crucial for adaptation to iron starvation and virulence in Aspergillus fumigatus
Source: Virulence. 2019 Nov 6;10(1):925–34. doi: 10.1080/21505594.2019.1682760 (PMC6844326; doi:10.1080/21505594.2019.1682760)
Supplement: Supplemental Material [file kvir-10-01-1682760-s001.zip › Suppl tables.docx]

**The leucine biosynthetic pathway is crucial for adaptation to iron starvation and virulence in *Aspergillus fumigatus***

**Supplementary information**

| Strain | Genotype | Ref. |
| --- | --- | --- |
| wt, A1160P+ | A1160, ∆*akuB::pyrG+* | [[29](#_ENREF_29)] |
| ∆*leuA* | A1160P+, ∆*leuA::hph* | this study |
| ∆*leuC* | A1160P+, ∆*leuC::hph* | this study |
| *leuA^rec^* | A1160P+, ∆*leuA::hph*, pleuA^rec^, *ptrA* | this study |
| *leuC^rec^* | A1160P+, ∆*leuC::hph*, pleuC^rec^, *ptrA* | this study |

**Table S1** Strains used in this study

**Table S2** Primers used in this study. Add-on enzyme restriction sites are marked in red.

| Primer name | Primer sequence 5' - 3' | Gene |
| --- | --- | --- |
| For gene deletion | |  |
| leuA5'FW+ | AATTCGAGCTCGGTACAGTTGCAGATTCAATCGG | *leuA* 5' flank |
| leuA5'RV+ | CTAGGTTAGAGGGGCAAAGGCGA | *leuA* 5' flank |
| leuA3'FW+ | CTAGAAGTATGTCTCTATGCCGTGA | *leuA* 3' flank |
| leuA3'RV+ | GCCAAGCTTGCATGCCGCACATCTGCTTCACC | *leuA* 3' flank |
| leuAhphFW | TGCCCCTCTAACCTAGGTACAGAAGTC | *hph* |
| leuAhphRV | AGAGACATACTTCTAGAAAGAAGGATTAC | *hph* |
| TO3 | AATTCGAGCTCGGTACACGAAGCCGAGATTATG | *leuC* 5' flank |
| TO4 | ATAGGCATGGGAAAGGTAGAGAAAGT | *leuC* 5' flank |
| leuC3'FW+ | CTAGAAGTTCGCCTGTGTGTTTTAT | *leuC* 3' flank |
| leuC3'RV+ | GCCAAGCTTGCATGCCCCCACCCATCAGCCG | *leuC* 3' flank |
| TO15 | CCTTTCCCTAACCTAGGTACAGAAGTC | *hph* |
| leuChphRV | ACAGGCGAACTTCTAGAAAGAAGGATTAC | *hph* |
| leuA5'FW | AGTTGCAGATTCAATCGGA | *leuA* 5' flank |
| leuA3'RV | GCACATCTGCTTCACCC | *leuA* 3' flank |
| TO14 | ACGAAGCCGAGATTATG | *leuC* 5' flank |
| leuC3'RV | CCCACCCATCAGCCGC | *leuC* 3' flank |
| ohph14 | GAAGATGTTGGCGACCTC | *hph* |
| ohph15 | GAGAGCCTGACCTATTGC | *hph* |
| For gene reconstitution | |  |
| TO52 | GGAATT-CATATG-TGTAGTTGCAGATTCAATCG | *leuA* 5' flank |
| TO53 | CCTTGA-AAGCTT-TGCACATCTGCTTCACCC | *leuA* 3' flank |
| TO54 | GAATTT-GCGGCCGC-ACGAAGCCGAGATTATGTC | *leuC* 5' flank |
| TO55 | CAAGGC-ACTAGT-CCCACCCATCAGCCGC | *leuC* 3' flank |
| For Southern blot analysis | |  |
| TO6 | TCCTGCTTGTTGGATATGC | *leuA* 5' flank |
| TO7 | CTCCGGTACCGGCTAAC | *leuA* 5' flank |
| TO8 | ACATTGACTCTGCAAATAAAG | *leuA* 3' flank |
| TO9 | TTCTTCCGGAAAGAGCAAC | *leuA* 3' flank |
| TO10 | AACATCTTTCCAGCTTACTG | *leuC* 5' flank |
| TO11 | GGGAAAGGTAGAGAAAGTC | *leuC* 5' flank |
| TO12 | CTCTCTGGTTCGATTTTGG | *leuC* 3' flank |
| TO13 | TGTTTCTAGGCTGATCTATC | *leuC* 3' flank |
| For Northern blot analysis | |  |
| oAf_LeuB_neu_F | CTGGACTATCATCAGCGTTG | *leuB* |
| oAf_LeuB_neu_R | GTCAAACAAGAGCGACATGC | *leuB* |
| LeuC_F | TGGCTGGCTACTGATCTG | *leuC* |
| LeuC_R | TCGACTTGAGGTGGTACG | *leuC* |
| Leu2A_F | GACGAAGCTCTTAACGCC | *leu2A* |
| Leu2A_R | CTCGATCACATTGCGGAC | *leu2A* |
| TO 86 | GATCAACCGTGGTTACCG | *gdhA* |
| TO 87 | ACCACCAGTCACCCTGC | *gdhA* |
| oAfmirB1me | AAGCCGAGAAAAAGGGGG | *mirB* |
| oAfmirB2me | AACCCAGATGAAGCCCAG | *mirB* |
| MSD1 | AACTACCTCCACCAGAAG | *sidA* |
| MSD2 | GAACGGCAATGTTGTAAG | *sidA* |
| oHapX-seq1 | TCGGTGGAAAGAAGTGCC | *hapX* |
| oAfhapXseq.r | CGACGATGTATTGTTATTGG | *hapX* |
| gap1fwd | ATGGAGAAAGATTCTATTGAACCTAAG | *gap1* |
| gap1rev | TATGCGAGGAATTGAGGTGC | *gap1* |

**Table S3** Homologies of *S. cerevisiae* leucine biosynthesis proteins to *A. fumigatus* proteins.

|  | *S. cerevisiae* | | | *A. fumigatus* | | BLAST | |
| --- | --- | --- | --- | --- | --- | --- | --- |
| enzyme | protein name | systematic name | protein name | | systematic name | E value | identity |
| acetohydroxy acid synthase | Ilv2p | YMR108W | Ilv2 | | AFUB_038840 | 0.0 | 419/668 (63%) |
| acetohydroxy acid reductoisomerase | Ilv5p | YLR355C | Ilv5 | | AFUB_034740 | 0.0 | 279/388 (72%) |
| dihydroxy acid dehydratase | Ilv3p | YJR016C | Ilv3A | | AFUB_029830 | 0.0 | 369/590 (63%) |
|  |  |  | Ilv3B | | AFUB_003960 | 0.0 | 301/543 (55%) |
|  |  |  | Ilv3C | | AFUB_007680 | 1e-72 | 174/563 (31%) |
|  |  |  | Ilv3D | | AFUB_031970 | 4e-58 | 152/531 (29%) |
| α-IPM synthase | Leu4p | YNL104C | LeuC | | AFUB_014560 | 0.0 | 340/586 (58%) |
|  | Leu9p | YOR108W |  |  |  | 0.0 | 329/587 (56%) |
| α-IPM isomerase | Leu1p | YGL009C | LeuA | | AFUB_027020 | 0.0 | 481/754 (64%) |
| β-IPM dehydrogenase | Leu2p | YCL018W | Leu2A | | AFUB_015310 | 2e-178 | 236/360 (66%) |
|  |  |  | Leu2B | | AFUB_001150 | 7e-119 | 186/364 (51%) |
| branched-chain amino acid amino transferase | Bat2p | YJR148W | Bat2 | | AFUB_063280 | 2e-168 | 222/362 (61%) |

**Figure S1. Overview of leucine biosynthesis in *S. cerevisiae***

**Figure S2. Deletion strategy for *leuA* and *leuC.* [A]** Schematic overview of the bipartite marker technique used for deletion of *leuA* and *leuC.* **[B]** and **[C]** Genomic organization of the *leuA* and *leuC* locus in the wt, the respective deletion mutants and the respective reconstituted strains*.*

**Figure S3. Supplementation with α- IPM or β-IPM does not rescue growth of the ∆*leuA* or ∆*leuC* mutant.** 10^3^ spores of *A. fumigatus* wt, ∆*leuA* or ∆*leuC* were point-inoculated on minimal medium containing 1 - 5 mM of α- IPM or β-IPM. Supplementation with 5 mM leucine served as growth control.

**Figure S4. Reintegration of the *leuC* gene (strain *leuC^rec^*) cures the defect in regulation of LeuB target genes and expression of *gap1* is not affected by lack of LeuC.** For Northern blot analysis, fungal strains were cultured under iron starvation conditions with 5 mM leucine supplementation. wt, *leuA^rec^*, *leuC^rec^* strains were grown for 16 h, ∆*leuA* for 20 h, and ∆*leuC* for 28 h to compensate for the different growth rate and to reach the same biomass.
