# Supplementary figures and images for: The leucine biosynthetic pathway is crucial for adaptation to iron starvation and virulence in Aspergillus fumigatus
Source: Virulence. 2019 Nov 6;10(1):925–34. doi: 10.1080/21505594.2019.1682760 (PMC6844326; doi:10.1080/21505594.2019.1682760)

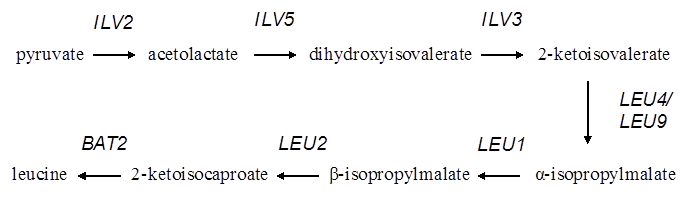

Supplement: Supplemental Material [file kvir-10-01-1682760-s001.zip › Figure S1.tif]

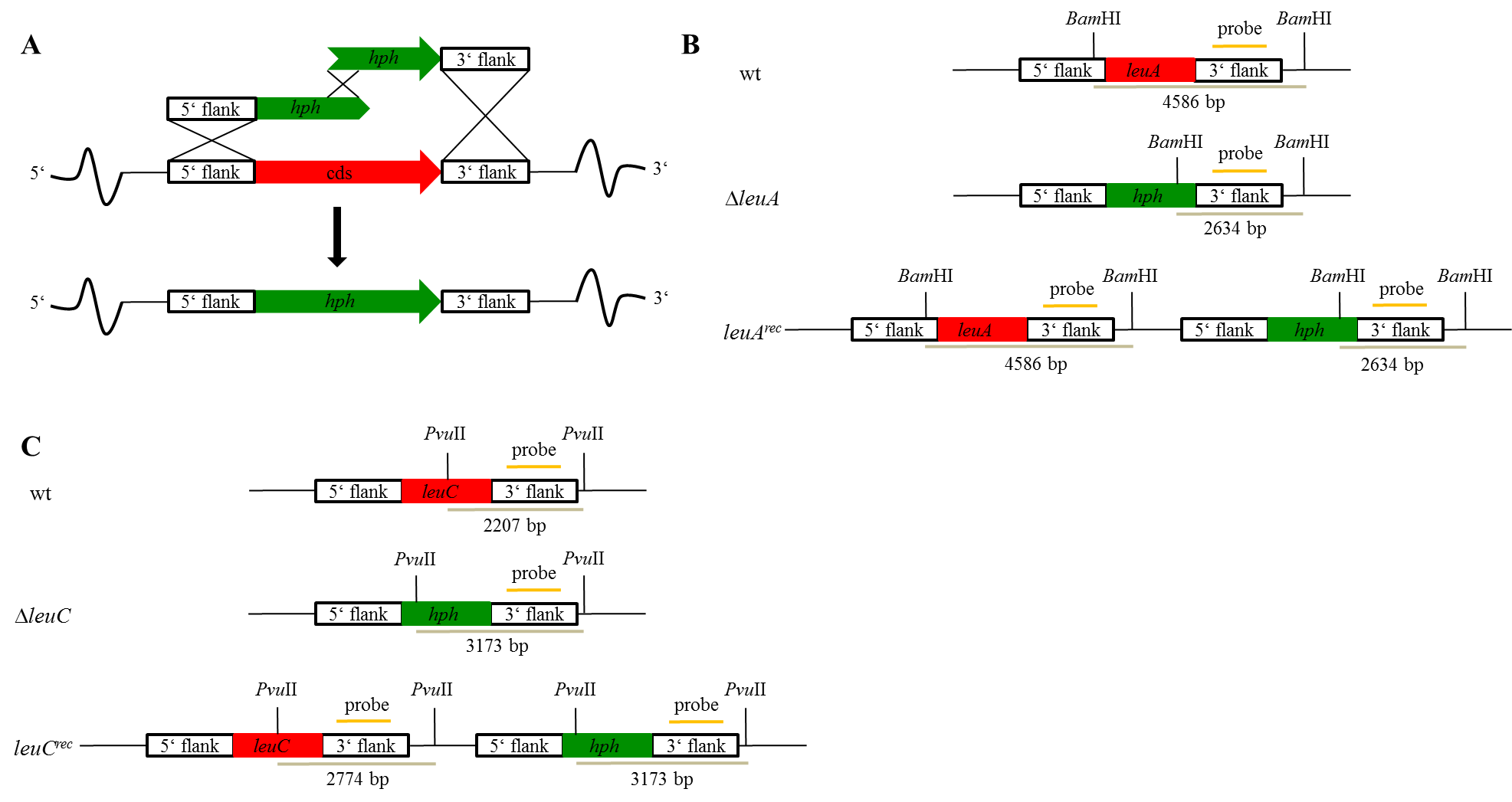

Supplement: Supplemental Material [file kvir-10-01-1682760-s001.zip › Figure S2.tif]

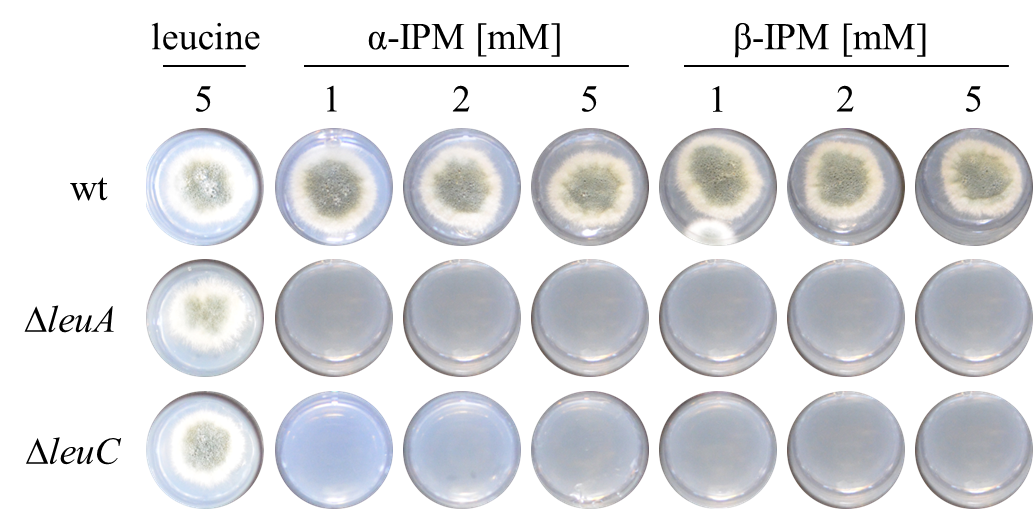

Supplement: Supplemental Material [file kvir-10-01-1682760-s001.zip › Figure S3.tif]

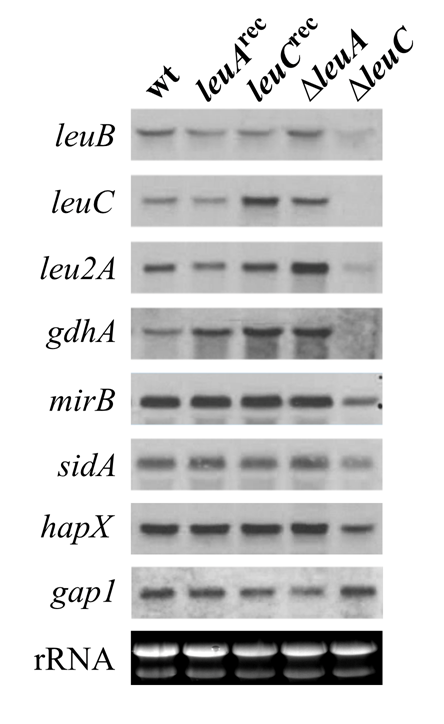

Supplement: Supplemental Material [file kvir-10-01-1682760-s001.zip › Figure S4.tif]
